# Towards standardized patient reported physical function outcome reporting: linking ten commonly used questionnaires to a common metric

**DOI:** 10.1007/s11136-018-2007-0

**Published:** 2018-10-13

**Authors:** M. A. H. Oude Voshaar, H. E. Vonkeman, D. Courvoisier, A. Finckh, L. Gossec, Y. Y. Leung, K. Michaud, G. Pinheiro, E. Soriano, N. Wulfraat, A. Zink, M. A. F. J. van de Laar

**Affiliations:** 1 Department of Psychology, Health and Technology, University of Twente, PO BOX 50 000, 7500 KA Enschede, The Netherlands; 2 Arthritis Center Twente and Department of Rheumatology and Clinical Immunology, Medisch Spectrum Twente, Enschede, The Netherlands; 3 Division of Rheumatology, University Hospitals of Geneva, Geneva, Switzerland; 4 UPMC Univ Paris 06, GRC-UPMC 08, Paris, France; 5 Rheumatology Department, Pitié Salpêtrière Hospital, APHP, Paris, France; 6 Department of Rheumatology and Immunology, Singapore General Hosptial, Singapore, Singapore; 7 Duke-NUS Medical School, Singapore, Singapore; 8 The National Databank for Rheumatic Diseases, Wichita, KS and University of Nebraska Medical Center, Omaha, NE USA; 9 Discipline of Rheumatology, Universidade do Estado do Rio de Janeiro, Rio de Janeiro, Brazil; 10 Rheumatology Unit, Internal Medical Services, Hospital Italiano de Buenos Aires, and Instituto Universitario Hospital Italiano de Buenos Aires, Buenos Aires, Argentina; 11 Wilhelmina Children’s Hospital, Utrecht, The Netherlands; 12 German Rheumatism Research Centre, Epidemiology Unit, and Rheumatology and Clinical Immunology, Charité University Medicine Berlin, Berlin, Germany

**Keywords:** Common metric, Item response theory, Physical function, Patient reported outcomes, Item bank

## Abstract

**Objectives:**

Outcomes obtained using different physical function patient reported outcome measures (PROMs) are difficult to compare. To facilitate standardization of physical function outcome measurement and reporting we developed an item response theory (IRT) based standardized physical function score metric for ten commonly used physical function PROMs.

**Methods:**

Data of a total of 16,386 respondents from representative cohorts of patients with rheumatic diseases as well as the Dutch general population were used to map the items of ten commonly used physical function PROMs on a continuous latent physical function variable. The resulting IRT based common metric was cross-validated in an independent dataset of 243 patients with gout, osteoarthritis or polymyalgia in which four of the linked PROMs were administered.

**Results:**

Our analyses supported that all 97 items of the ten included PROMs relate to a single underlying physical function variable and that responses to each item could be described by the generalized partial credit IRT model. In the cross-validation analyses we found congruent mean scores for four different PROMs when the IRT based scoring procedures were used.

**Conclusions:**

We showed that the standardized physical function score metric developed in this study can be used to facilitate standardized reporting of physical function outcomes for ten commonly used make physical function PROMs.

**Electronic supplementary material:**

The online version of this article (10.1007/s11136-018-2007-0) contains supplementary material, which is available to authorized users.

Physical function is an important indicator of the impact of disease on the daily lives of people living with medical conditions. Patient reported outcome measures (PROMs) of physical function are standardized questionnaires that ask patients to rate the difficulty they experience in performing a series of everyday tasks that require physical movement and exertion. Regulatory bodies consider PROMs the standard to support drug approval or labeling claims based on subjective endpoints in clinical trials. Accordingly, physical function PROMs are routinely used to evaluate therapeutic interventions in various medical conditions [1, 2]. Physical function PROMs are also commonly collected in patient registries to provide complementary, “real-world” information on patient outcomes for decision making by various stakeholders, including patients, healthcare providers, payers, and clinicians [3, 4].

Unfortunately, many different physical function PROMs are in widespread use, which makes outcomes difficult to compare between different data sources. This is because the individual PROM item scores are usually summed to characterize a patient’s level of physical function. A drawback of this approach is that the same patient may achieve different summed scores, depending on the characteristics of the items which he or she responded to. Therefore, if two groups of patients are assessed using different physical function PROMs, any observed summed score differences between the groups could be due to the groups of patients differing in physical function or due to one scale asking about activities that are on average more difficult to perform. In practice, this problem is usually circumvented by administering the same PROM to all patients. However, this limits the potential for secondary use of previously collected data and makes it difficult to develop data collection standards. Moreover, individual item responses are frequently missing, in which case the summed score provides a misleading summary of a patient’s level of physical function.

Item response theory (IRT) is a psychometric framework in which the relationship between observed item response behavior and the underlying variable measured by the PROM is mathematically described. The application of item response models allows a latent variable score of patients to be estimated from responses to any items that are calibrated to a common IRT scale. Therefore, when a collection of items is in a common IRT metric, different subsets of these items administered to the same patient should yield the same IRT score and IRT score differences between groups of patients who have responded to different PROMs can be meaningfully compared [5]. This has been illustrated in several previous studies which have found that IRT based scoring procedures yield congruent scores for different PROMs administered to a single group of patients [6–10].

In the present study we set out to develop a standardized IRT based reporting metric for physical function by calibrating ten commonly used physical function PROMs to a common IRT metric [11–20]. A secondary objective of the study was to cross-validate the common reporting metric in an independent dataset.

## Methods

### Data sources and selection

Data of patients with inflammatory rheumatic diseases were taken from the Swiss Clinical Quality Management (SCQM) registry [21], the United States National Data Bank of Rheumatic Diseases [22], The National Database of the German Collaborative Arthritis Centres (NDG) [23], and the Dutch Rheumatoid Arthritis Monitoring (DREAM) Study [24]. Data of pediatric patients with juvenile idiopathic arthritis were available from the Pharmachild registry [25]. For the Rasch Everyday Activity Limitations item bank, data were available from a sample of DREAM patients as well as a larger sample of 1128 people representative of the Dutch general population. [26]. Data were also used from a calibration study of PROMIS physical function item bank in Dutch RA patients [27]. In that longitudinal study, subsets of the items were administered to different patients at each time point. Non-IRT analyses that require a complete data matrix could therefore not be performed for the PROMIS data. Finally data of the Psoriatic Arthritis Impact of Disease study were used [28].

To ensure that all included PROMs assess the same or a highly similar construct, we selected PROMs from the available datasets that met the following criteria: (1) the PROM is commonly referred to in the literature as a measure of physical function/activity limitations, (2) all of its items assess the level of difficulty experienced in performing everyday tasks that require physical movement and/or exertion, and (3) the PROM is not limited to assessing the functioning of specific body parts or intended to be used in a particular, specified patient population. Descriptive information about the PROMs that met these criteria is presented in Table 1. Three EQ-5D items that met the content criterion were also included. Patients who were administered all PROMs included in a dataset were selected for analysis.

**Table 1:** Characteristics of included physical function measures

Scale	Abbreviation	*N*	Items	Response options
Bath Ankylosing Spondylitis Functional Index (BASFI)	BASFI	2839	10	11
Childhood Health Assessment Questionnaire	CHAQ	1029	30	4
Funktionsfragebogen Hannover	FFbH	4201	18	3
Health Assessment Questionnaire	HAQ-DI	9913	20	4
Health Assessment Questionnaire Two	HAQ-II	6538	10	4
Modified Health Assessment Questionnaire	MHAQ	6538	8	4
Numerical Rating Scale	NRS	5403	1	11
PROMIS Short Form v2.0—Physical Function 10a	PROMIS	699	10	5
Rasch Everyday Activity Limitations 10	REAL-10	1377	10	5
The Short Form (36) Health Survey Physical Functioning	SF-36 PF10	5975	10	3

*N* = total number of patients included in the present study who filled out the questionnaire

### Analysis

Preceding the analyses, all items were recoded so that higher scores indicate better functioning and item response options were collapsed if they had attracted < 20 responses.

#### Checking the assumption of monotonicity

In parametric IRT models for ordered polytomous data, the expected item scores are constrained to be monotonically increasing over the latent variable. The first step of the analysis was to examine whether the observed items scores also increased monotonically with the observed score. This was achieved by inspecting non-parametric, kernel smoothed plots of the average item scores across the respective observed score continua, using the KernSmoothIRT R package [29].

#### Essential uni-dimensionality

It is further assumed that a single latent variable explains how patients respond to an item. This assumption can be supported by showing that a measurement model with a single underlying factor can sufficiently account for the variance in item scores. This assumptions was tested using confirmatory factor analysis (CFA) using the mean and variance adjusted weighted least squares estimator in MPLUS [30]. BASFI and NRS items were considered continuous variables, because items with > 10 response options could not be specified as categorical in MPLUS. The remaining items were considered categorical variables, so that the correlation matrices of datasets with NRS or BASFI items included a mixture of polychoric, polyserial, and Pearson’s correlations, depending on the involved items. Two datasets (SCQM and NDG) had to be split to obtain complete data matrices for this analysis because different sets of items were presented to different patients. The Tucker Lewis Index (TLI), Comparative fit index (CFI), and the root mean square error of approximation (RMSEA) were used to judge goodness of fit [31, 32]. We used conventional cut-off values for these indices (CFI/TLI ≥ 0.95 and RMSEA < 0.08).

The degree to which the item response data could be explained by a single, dominant latent variable was further explored using hierarchical exploratory factor analyses on the Schmid Leiman transformed factor matrices, with the R psych package [6, 33, 34]. In these analyses, each item included in a dataset loaded on one general factor (i.e., physical function) as well as one of three “group” factors, which represent covariance among subsets of items unaccounted for by the general factor. We decided to extract three factors, since it is the minimum number of extracted factors recommend by the Psych package authors and the ECV’s and Omega coefficients proved to be relatively insensitive to increases in the number of extracted factors. Since for the purpose of our analysis, the substantive meaning of the group factors was less important than their combined magnitude, these group factors were not specified a priori, but extracted using the maximum likelihood estimator. We obtained McDonald’s hierarchical omega coefficients to estimate the general factor saturation of each data set, as well as the Explained Common Variance (ECV), which is the ratio of the general factor eigen value to the sum of all four eigen values. Both statistics are measures of the strength of the general factor relative to the group factors and higher values provide stronger support for the essential uni-dimensionality of the item response data. We used previously recommended cut-off values of 0.70 for coefficient omega and 0.60 for ECV to judge the appropriateness of a unidimensional measurement model [35].

#### IRT calibration of the item responses

The concurrent calibration method was used to simultaneously place the items on a common scale. This method involves combining all the datasets in a single item by person matrix, and setting the unobserved item responses missing. The items are then jointly calibrated. To achieve a common scale for all concurrently calibrated items, the different datasets need to be linked by common items [36] or by assuming a common distribution of physical function scores that applies to multiple datasets [37]. This latter assumption is usually only appropriate if different items are administered at random to patients from a specific population. Since we relied on previously collected data, the datasets were linked using anchor items that feature in > 1 dataset. Such a calibration design can be referred to as a non-equivalent group, common items design. Supplemental Fig. 1 presents an overview of the anchors between datasets. Previous simulation studies found that accurate parameter estimates can be obtained using this method for ordered polytomous data, even when the percentage of common items is low [38, 39]. The marginal maximum likelihood estimator, with dataset specific score distributions was used to obtain estimates of the item parameters and the means and standard deviations of the populations [40].

After item parameters had been obtained for 9 out of 10 PROMs, the Stocking-Lord (SL) method was used to link the item parameters of the PROMIS Short Form v2.0—Physical Function 10a to the obtained common scale [41]. The SL method allowed us to rescale the item parameters for the PROMIS short form using the item parameters which where previously obtained in a Dutch calibration study, since the Sf-36 physical functioning scale and the HAQ-DI were also calibrated in that study.

#### Model fit

We compared the fit of the Rasch based partial credit model and a two parameter generalization, the generalized partial credit model using a likelihood ratio test for nested models and the Akaike information criterion (AIC) which is a model selection criterion that penalizes models for their number of parameters [42, 43]. Item fit and differential item functioning (DIF) across data sets and (patient) populations was evaluated using a Lagrange Multiplier statistic and associated effect size statistic [44]. Because these statistics have been shown to have high “false alarm” rates as sample size goes up [45], items were flagged for DIF or lack of fit in case of a significant LM test and $E{S_{DIF}}$ > ± 0.05, as recommended by Glas and Falcón. DIF affected items were assigned subgroup item parameters [46].

#### Checking for deviations of local independence

A final IRT assumption is that the associations between items are fully explained by the latent variable. In real data, this assumption is usually violated. Local deviations of this assumption were analyzed in the matrix of residuals using Yen’s Q3 statistics. Items were flagged for local dependence (LD) if Q3 > 0.25, which corresponding to 6.3% shared variance between a pair of residuals [5, 47].

### Psychometric properties of linked scores

Global reliability of the IRT scores for each PROM was evaluated by obtaining marginal reliability coefficients [48]. The reliability of IRT scores at different locations of the latent variable was evaluated using conditional reliability coefficients [49]. Reliability of scores obtained using the traditional scoring procedures was also evaluated, using greatest lower bound reliability coefficients [50]. We obtained Pearson’s correlations between the observed scores and IRT scores for each PROM. Since both scoring procedures summarize the same information, the correlation should be extremely strong (*r* > 0.95). Furthermore, if all scales assess a single latent variable, the correlations between their summed scores should be strong (*r* > 0.70). For the inter-scale correlations, a correction procedure was employed in case more than one item was shared between scales to adjust the correlations for spurious inflation, due to items being included in multiple PROMs [51]. In cases where all items from one PROM were included in another (i.e., a short form), Levy’s corrected correlation coefficients were obtained [52].

### Assessment of the common metric in independent data

Next we examined if and to what extent standardized physical function scores obtained using different PROMs were congruent. For this analysis we used data of a previous study in which four different PROMs included in the common metric were administered to a group of 243 consecutive patients with gout, osteoarthritis, or polymyalgia visiting the rheumatology clinic of Medisch Spectrum Twente. Patient characteristic are described in Supplemental Table 1 and further details about the study are provided elsewhere [53]. Patients who filled out each of the PROMs were selected for analysis, including those with missing responses for some PROMs. Standardized physical function scores were estimated using the Expected a posteriori method. Since IRT scores should be less dependent on the specific items that were used, we expected that the means of the IRT scores for different PROMs would not be significantly different and effect sizes of trivial magnitude (*ES* < 0.20) would be found for all comparisons. We compared the obtained results with unadjusted summed scores, rescaled to range from 0 to 100.

## Results

Table 2 presents characteristics of the included samples. Data of 16,863 respondents were used to estimate the item parameters. Mean standardized physcial function scores were similar for the various inflammatory arthritis cohorts and clearly higher for the Dutch general population sample. More detailed descriptions of the individual datasets are provided in the Supplemental Material.

**Table 2:** Respondent characteristics

	DREAM *N* = 941	LISS *N* = 1128	NDB *N* = 6961	NDG *N* = 4201	Pharmachild *N* = 1029	PSAID *N* = 474	SCQM *N* = 3157
Females, *n* (%)	591 (62.8%)	604 (53.5%)	5388 (77.4%)	2960 (70.5)	775 (64.9%)	235 (49.6%)	1669 (52.9%)
Age in years, mean (SD)	57.23 (11.75)	50.36 (17.99)	60.60 (12.48)	61.48 (13.95)	16.03 (4.76)	50.38 (12.69)	46.60 (14.20)
Linked score, mean (SD)	69.30 (13.53)	82.90 (16.94)	65.35 (13.33)	69.69 (13.68)	77.88 (11.86)	66.83 (13.85)	65.67 (11.97)

*DREAM* Dutch Rheumatology Monitoring Study, *LISS* longitudinal internet studies for the social sciences, *NDB* United States National Data Bank of Rheumatic Diseases, *NDG* The National Database of the German Collaborative Arthritis Centre, *PSAID* psoriatic arthritis impact of disease study, *SCQM* Swiss Clinical Quality Management registry

### IRT assumptions

#### Checking the assumption of monotonicity

Preceding the psychometric analysis, we collapsed the extreme response options (“With much difficulty” and “unable to do”) for 3 PROMIS items (PFB26, PFA55, and PFC45r1) and CHAQ items 4–8, due to lack of data. The expected item scores were strictly increasing for all items. We concluded that none of the items showed violations of the expected form of the item characteristic curves.

#### Essential uni-dimensionality

The results of the analysis of essential uni-dimensionality are summarized in Table 3. For seven of the datasets, all fit indices indicated sufficient goodness of fit of a unidimensional model, with a strong general factor and generally small eigenvalues for the three group factors according to the results of the hierarchical factor analysis. For the Pharmachild data, the RMSEA was slightly above the threshold of 0.08 and for one of the NDG samples, the one-dimensional model was rejected by all CFA fit indices. However, in both cases a clear dominant factor was found in the hierarchical factor analysis, with omega > 0.70 and ECV of the general factor > 0.60. Inspection of the factor loadings did not reveal a clear pattern with respect to the type of items that loaded on the group factors. Our overall conclusion was that the response data were essentially uni-dimensional in all datasets.

**Table 3:** Essential uni-dimensionality of the individual datasets

Dataset	PROMs	CFI	TLI	RMSEA	Coefficient omega	ECV	ECV NF
DREAM	HAQ-DI, PF10, REAL-10	0.98	0.98	0.08	0.78	0.66	0.02–0.21
LISS	PF10, REAL-10	0.99	0.99	0.06	0.72	0.60	0.05–0.26
NDB	HAQ-DI, PF10, HAQ-II	0.99	0.99	0.04	0.87	0.81	0.00–0.08
NDG	NRS, EQ-5D, FfbH	0.99	0.99	0.04	0.83	0.74	0.01–0.11
NDG	BASFI, EQ-5D, NRS, FfbH	0.90	0.89	0.14	0.85	0.80	0.03–0.08
Pharmachild	HAQ-DI, C-HAQ	0.98	0.97	0.09	0.80	0.70	0.00–0.13
PSAID	HAQ-DI EQ-5D, PF10, NRS	0.99	0.99	0.06	0.78	0.68	0.05–0.16
SCQM AS	BASFI, EQ-5D, PF10	0.98	0.98	0.07	0.80	0.70	0.06–0.13
SCQM _RA	HAQ-DI Eq-5D, PF10	0.99	0.99	0.06	0.86	0.75	0.02–0.08

*CFI* Comparative Fit Index, *TLI* Tucker Lewis Index, *RMSEA* root mean sqaured error of approximation, *ECV* explained common variance, *ECV NF* explained common variance nuisance factors, *DREAM* Dutch Rheumatology Monitoring Study, *LISS* longitudinal internet studies for the social sciences, *NDB* United States National Data Bank of Rheumatic Diseases, *NDG* The National Database of the German Collaborative Arthritis Centre, *PSAID* psoriatic arthritis impact of disease study, *SCQM* Swiss Clinical Quality Management registry, *BASFI* Bath Ankylosing Spondylitis Function Index, *CHAQ* Childhood Health Assessment PROM, *FFbH* Funktionsfragebogen Hannover, *HAQ-DI* Health Assessment PROM Disability Index, *HAQ-II* Health Assessment PROM Two, *MHAQ* Modified Health Assessment PROM, *NRS* numerical rating scale, *PROMIS* Patient Reported Outcomes Measurement Information System Short Form v1.0—Physical, *REAL-10* Rasch Everyday Activity Limitations Item Bank Short Form 10

### IRT item fit and differential item functioning

We used the two parameter generalized partial credit model for item calibration, because this model fitted the data better according to the results of the likelihood ratio test (${\chi ^2}$ = 31,014, *p* ≤ 0.01) as well as according to AIC (${\Delta _{{\text{AIC}}}}$ = 30,862). In the analysis of DIF across datasets, HAQ item 11 was flagged (*LM* = 547.29, *p* < 0.01, $E{S_{{\text{DIF}}}}$ = 0.06), as were BASFI items 2 (*LM* = 35.59, *p* < 0.01, $E{S_{{\text{DIF}}}}$ = 0.05) and 8 (*LM* = 78.44, *p* < 0.01, $E{S_{{\text{DIF}}}}$ = 0.05). Scores were lower than expected in all datasets except the US dataset. For both BASFI items, scores were lower than expected in PSA patients. US (HAQ 11) and PSA (BASFI items) specific item parameters were therefore assigned and fit of the re-specified model was examined. Only 6 (2%) of the items met the criteria for misfit. None of these items showed lack of fit in more than one dataset.

### Local independence

Examination of the matrix of residuals revealed that 10% of the item pairs had Q3 > ± 0.25, suggestive of local dependence. SF36 Items 4 (31%) (Walking more than a mile), and 7 (31%) and 8 (33%) (Climbing stairs), as well as seven out of ten REAL items were flagged particularly often. To explore the impact of LD on the item parameter estimates, we re-ran the analysis with these items removed and compared the item parameters for the remaining items with those obtained in the initial run. The Pearson’s correlation between the re-estimated item parameters and the original ones was near perfect (*r* > 0.99) for both the discrimination and threshold parameters. Nevertheless, most item parameters were now slightly different, with root mean squared deviations of 0.05 and 0.09 for the discrimination and threshold parameters respectively. However, these changes did not result in noticeably different predicted response probabilities for any of the items, which is illustrated in Supplemental Fig. 2 for the item for which the item parameters had changed the most (SF-36 item 10).

### Stocking-Lord linking of PROMIS items

**Fig. 1:**
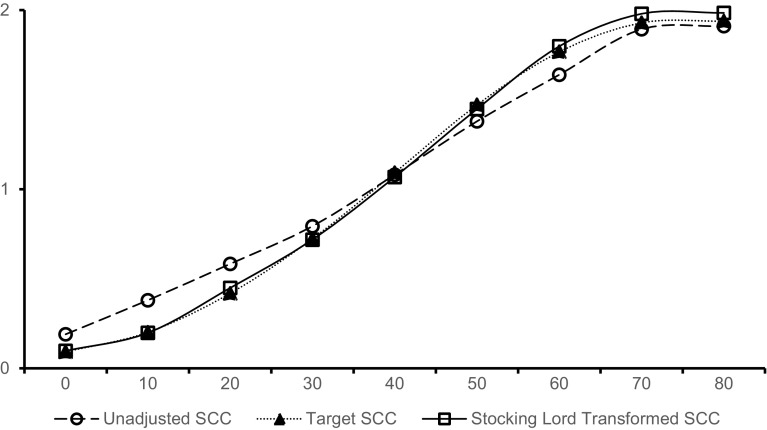
Scale characteristic curve, mapping raw summed score to IRT scores for SF-36 PF-10 obtained from Dutch RA patients before (gray line) and after (straight black line) Stocking-Lord transformation, compared with scale characteristic curve obtained in the concurrent calibration (dashed black line). *SCC* scale characteristic curve

To illustrate the results of the Stocking-Lord rescaling procedure employed to link PROMIS items to the common scale, Fig. 1 presents a mapping of the observed scores on the IRT metric for different calibrations of the SF-36 PF-10 items, which served as the anchor in that data set. For most of the observed score levels, the corresponding IRT scores differ noticeably between the IRT calibrations of the Dutch RA patients (*n* = 691, gray solid line in Fig. 1) and the results of the concurrent calibration in the present study (dotted black line in Fig. 1). After transforming the item parameters using the Stocking-Lord linking coefficients, these differences disappeared almost completely (solid black line in Fig. 1), which supports the conclusion that the transformed PROMIS item parameters are on the same scale as the item parameters of the other PROMs included in the common metric.

### Standadized physical function score metric

**Fig. 2:**
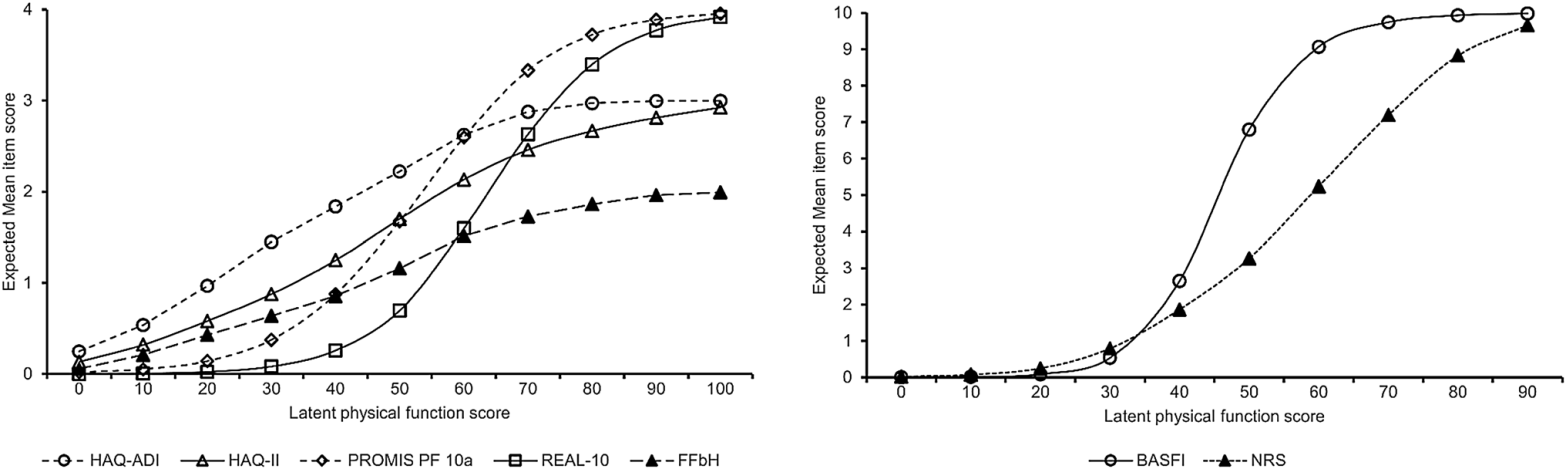
Mapping of in individual physical function PROM mean expected scores on the standardized physical function score metric. *HAQ-ADI* Health Assessment Questionnaires Disability Index, *HAQ-II* Health Assessment Questionnaires Disability Index Two, *PROMIS* patient reported outcomes measurement information system, *REAL* Rasch assessment of everyday activity limitations, *FFbH* Funktionsfragenbogen Hannover, BASFI Bath Ankylosing Spondylitis Functional Index, *NRS* numerical rating scale

Figure 2 plots the expected mean item scores for individual physical function PROMs across different levels of the standardized physical function score metric. Higher standardized physical function scores indicate a higher level of physical functioning. The score metric was scaled so that a standardized score of 0 corresponds with an expected mean item score of 0 for each of the physical function PROMs that have been linked to the standardized physical function score metric. That is, a score of zero represents a lower bound of physical function levels that can be measured using the included PROMS. Increments of 10 points on the standardized physical function score metric correspond with increments of 1 point on the underlying IRT logit scale. The mean standardized physical function score in the calibration sample was 70 (*SD* = 10).

### Psychometric properties of the included physical function PROMs

Table 4 provides a summary of characteristics of the ten included physical function PROMs. As expected, the correlations between the observed scores and IRT scores for individual PROMs approached one for all PROMs except the NRS, which was the only score obtained using a single item. The overlap corrected correlations between scales were all > 0.70, further supporting the earlier conclusion that all PROMs assess a similar latent variable. Scores of all scales were highly reliable according to the global reliability coefficients and of similar magnitude for the IRT and observed scores.

**Table 4:** Psychometric properties of included scales

Scale	Inter scale correlations	Observed/IRT score correlation	GLB	Marginal reliability	Range of IRT scores for which *CR* > 0.70
BASFI	0.72–0.86	0.98	0.98	0.98	0–60
CHAQ	0.81–0.91	0.94	0.98	0.98	0–90
FFbH	0.72–0.75	0.98	0.97	0.97	0–80
HAQ-DI	0.76–0.92	0.97	0.97	0.95	0–80
HAQ-II	0.80–0.87	0.99	0.95	0.92	0–80
MHAQ	0.74–0.92	0.98	0.92	0.87	0–60
NRS	0.72–0.72	0.73	NA	NA	NA
PROMIS	NA	NA	NA	0.95	0–80
REAL-10	0.71–0.85	0.99	0.97	0.98	0–90
SF-36 PF10	0.71–0.81	0.98	0.96	0.94	0–80

*BASFI* Bath Ankylosing Spondylitis Function Index, *CHAQ* Childhood Health Assessment PROM, *FFbH* Funktionsfragebogen Hannover, *HAQ-DI* Health Assessment PROM Disability Index, *HAQ-II* Health Assessment PROM Two, *MHAQ* Modified Health Assessment PROM, *NRS* numerical rating scale, *PROMIS* Patient Reported Outcomes Measurement Information System Short Form v1.0—Physical, *REAL-10* Rasch Everyday Activity Limitations Item Bank Short Form 10, *GLB* greatest lower bound reliability coefficient, *CR* conditional reliability coefficients

### Accuracy of IRT scores for different PROMs in independent data

The results of the external validation exercise in which the IRT based scoring procedures were applied in an independent data set are summarized in Table 5. The standardized physical function scores for the four different PROMs were very similar for the different PROMs, with effect sizes of trivial magnitude for all comparisons. By comparison, the rescaled summed scores were quite different for the different PROMs, with effect sizes of moderate to large magnitude, highlighting the dependence of the scores on item characteristics.

**Table 5:** Agreement over IRT scores in independent dataset

	Observed scores* (SD)	Range of *p*-values**	Range of Cohen’s *D*’s	IRT scores (SD)	Range of *p*-values	Range of Cohen’s *D*’s
HAQ-ADI	29.09 (24.6)	< 0.01	0.71	56.36 (17.43)	0.19	0.09
HAQ-II	36.22 (22.61)	< 0.01–< 0.01	0.67–1.55	58.60 (16.50)	0.08–0.30	0.07–0.12
MHAQ	18.58 (19.06)	< 0.01–< 0.01	1.12–1.55	56.63 (16.36)	0.30–0.33	0.07–0.07
SF-36 PF10	48.22 (26.94)	< 0.01–< 0.01	1.12–1.55	57.46 (13.38)	0.08–0.33	0.07–0.12

*HAQ-DI* Health Assessment PROM Disability Index, *HAQ-II* Health Assessment PROM Two, *MHAQ* Modified Health Assessment

*Rescaled to range from 0 to 100

**Bonferroni corrected

## Discussion

In our present study, we have calibrated ten of the most commonly used physical function PROMs to a standardized physical function score metric, which allows physical function outcomes obtained using different measures to be reported in a unified metric. Standardized reporting of physical function outcomes is advantageous because it allows new and ongoing data collection initiatives (e.g., patients registries, clinical trials) to use or keep using their preferred physical function PROMs, while at the same time allowing the results to be compared to others that have chosen to use a different PROM. This increases the potential for secondary uses of already collected data, for example for comparative performance assessments, collaborative research projects, or systematic reviews. It may also ease the process of developing standards, including standardized datasets, for outcome measurement. For instance the standardized physical function score metric presented here will be used in the International Consortium for Health Outcomes Measurement (ICHOM) Standard set for inflammatory arthritis (see http://www.ichom.org) to allow comparisons of physical function outcomes obtained by healthcare providers in different healthcare systems.

The current study used an elaborate approach to link the items of 10 commonly used physical function PROMS to a single latent physical function scale. As part of this approach we considered the partial credit model and its two parameter generalization for item calibration, since this allowed us to examine whether a Rasch type model would be appropriate for the response data. We also explored the underlying assumptions of the IRT models for ordered polytomous models in detail. As a by-product of these analyses, our current results further support that each of the included PROMs essentially measures the same, single underlying construct and yields reliable scores. In a final step of the process we tested the performance of the common reporting metric and were able to demonstrate that congruent standardized physical function scores could be obtained from four different PROMs applied to a single group of patients. These IRT based score estimates are unique to each score pattern (i.e., likely to differ for patients with the same summed score) and require specific software or detailed knowledge of IRT to obtain. Researchers interested in using the standardized Physical function score metric may contact the corresponding author or upload sample summary statistics or anonymized patient level item response data at http://www.tihealthcare.nl to obtain standardized physical function scores.

Previously, the HAQ-DI and PF10 were linked to a common Rasch scale and both PROMs were linked to the PROMIS physical function metric as part of the PROSETTA project, using a two parameter IRT model [54, 55]. In these papers, crosswalk tables were provided for mapping the summed scores of one PROM into the metric of another. A limitation to crosswalk tables is that they pertain to the summed raw scores and can only be used in case there are no missing values. Moreover, crosswalk tables yield suboptimal score estimates for two parameter models, because not all information about a patient’s physical function level is provided by their summed scores. Further, both previous studies included a limited number of PROMs and, in the PROSETTA paper, a small convenience sample with a high average level of physical function was used. However, many of the items included in HAQ-DI and PROMIS target moderate to severe levels of physical disability. A strong point of our current study is that the item response models were estimated in several large, representative samples of different (patient) populations, with physical function levels that were well matched with the PROM items.

While the presented results are encouraging, and the finding that few items showed DIF was reassuring, all data currently used are from European or US patients and we exclusively used data from patients with inflammatory rheumatic conditions and the general population. Future studies are needed to examine invariance across different patient populations. Another limitation of the current version is that response options had to be collapsed for some of the items of PROMIS (PFB26, PFA55, & PFC45r1) and CHAQ (items 4–8) to obtain proper estimates for some of the item parameters, reducing the amount of information provided by the individual items to the overall score [56]. Furthermore, the bivariate associations involving BASFI and NRS items could have been underestimated because correlation coefficients that are considered appropriate for ordinal data, could not be used for these items in MPLUS or the R Psych package, because they have > 10 response options. As illustrated previously this could have led to a slight underestimation of model fit in the CFAs involving datasets with BASFI and NRS items [57]. We further found relatively large number of locally dependent items. However, this did not seem to have a major impact on the item parameter estimates, and previous studies suggest that LD of similar magnitudes have a negligible practical impact on equating results [58]. Finally, to ensure that score comparisons between the different PROMs included in the common metric would be meaningful, we used inclusion criteria aimed at ensuring that the included PROMs assess a similar construct. Nevertheless, previous studies have shown that their items cover a range of different health concepts, predominantly related to mobility, self-care, and the ability to tend to domestic responsibilities and do sports [59, 60]. We caution users of the common metric that the degree to which each of these facets of physical function is represented differs across the included PROMs.

In summary, this study reports on the development of a common metric for physical function which can be used for harmonizing physical function PROMs reporting by facilitating outcomes comparisons in settings in which different PROMs are used. Detailed instructions on how to use the common metric are provided on http://www.tihealthcare.nl.

## Electronic supplementary material

Below is the link to the electronic supplementary material.

Supplementary material 1 (DOCX 33 KB)
