# Supplementary material for: Towards standardized patient reported physical function outcome reporting: linking ten commonly used questionnaires to a common metric
Source: Qual Life Res. 2018 Oct 13;28(1):187–97. doi: 10.1007/s11136-018-2007-0 (PMC6339672; doi:10.1007/s11136-018-2007-0)
Supplement: Supplementary file 1 — Supplementary material 1 (DOCX 33 KB) [file 11136_2018_2007_MOESM1_ESM.docx]

| Dataset | HAQ-DI | SF-36 PF10 | REAL-10 | HAQ-II | NRS | EQ-5D | FfbH | C-HAQ | BASFI |
| --- | --- | --- | --- | --- | --- | --- | --- | --- | --- |
| DREAM |  |  |  |  |  |  |  |  |  |
| LISS |  |  |  |  |  |  |  |  |  |
| NDB |  |  |  |  |  |  |  |  |  |
| NDG |  |  |  |  |  |  |  |  |  |
| NDG |  |  |  |  |  |  |  |  |  |
| Pharmachild |  |  |  |  |  |  |  |  |  |
| PSAID |  |  |  |  |  |  |  |  |  |
| SCQM AS |  |  |  |  |  |  |  |  |  |
| SCQM _RA |  |  |  |  |  |  |  |  |  |

Supplemental figure 1 Linking design

BASFI = Bath Ankylosing Spondylitis Function Index; CHAQ = Childhood Health Assessment PROM; FFbH = Funktionsfragebogen Hannover; HAQ-DI = Health Assessment PROM Disability Index; HAQ-II = Health Assessment PROM Two; MHAQ = Modified Health Assessment PROM; NRS = Numerical Rating Scale; PROMIS = Patient Reported Outcomes Measurement Information System Short Form v1.0 – Physical; REAL-10 = Rasch Everyday Activity Limitations Item Bank Short Form 10.

Supplemental table 1 Dutch Rheumatology Monitoring Registry Sample characteristics ( N = 941)

| Females, n(%) | 591 (62.8%) |
| --- | --- |
| Age in years, Mean (SD) | 57.23 (11.75) |
| Linked score, Mean (SD) | 49.30 (13.53) |
| Disease Activity Score 28, Mean (SD) | 2.12 (1.1) |
| VAS Fatigue, Mean (SD) | 34.11 (29.97) |
| VAS Pain, Mean (SD) | 27.69 (23.46) |
| HAQ-DI, Mean (SD) | 0.54 (0.62) |

VAS = Visual analogue scale; HAQ-DI = Health Assessment Questionnaire disability Index

Supplemental table 2 Longitudinal Internet Studies for the Social sciences sample characteristics ( N = 1128)

| Age, years Mean (SD) | 50.36 (17.99) |
| --- | --- |
| Sex, n (%) |  |
| Male | 524 (46.5%) |
| Female | 604 (53.5%) |
| Educational level, n (%)* |  |
| Low | 314 (28%) |
| Middle | 416 (37%) |
| High | 394 (35%) |
| Occupational status, n (%) |  |
| Remuneratively employed | 546 (48.4%) |
| Pensioned | 261 (23.1%) |
| Student | 107 (9.5%) |
| Housekeeper | 85 (7.5%) |
| Looking for work | 54 (4.7%) |
| Unable to work | 41 (3.6%) |
| Voluntarily employed | 29 (2.6%) |
| Other | 5 0.4%) |
| Self-reported diagnosis of chronic condition, n (%) |  |
| Osteoarthritis | 162 (14.4%) |
| Diabetes mellitus | 65 (5.8%) |
| Asthma | 65 (5.8%) |
| COPD | 40 (3.5%) |
| Depression | 62 (5.5%) |
| Rheumatoid arthritis | 31 (3.0% |
| Fibromyalgia | (20 (1.8%) |
| Stroke | 6 (0.5%) |
| Hypertension | 211 (18.7%) |
| Migraine headaches | 59 (5.2%) |
| Any rheumatic condition | 190 (17%) |
| Any chronic condition | 491 (43.5%) |
| SF-36 Physical functioning, Mean (SD) | 83.06 (25.28) |

COPD = Chronic obstructive pulmonary disease; SF-36 = Short Form (36) Health Survey

Supplemental table 3 United States National Databank of Rheumatic Disease sample characteristics ( N = 6961)

| Females, n(%) | 5388 (77.4%) |
| --- | --- |
| Age in years, Mean (SD) | 60.60 (12.48) |
| BMI, Mean (SD | 27.06 (5.82) |
| Disease duration, in years, Mean (SD) | 14.88 (12.27) |
| Sf-36 Physical functioning, Mean (SD | 47.8 (27.90) |
| SF-36 Physical role limitations, Mean (SD | 41.58 (10.58) |
| SF-36 Bodily pain, Mean (SD | 31.15 (23.41) |
| SF-36 General Health, Mean (SD | 58.69 (9.70) |
| SF-36 Vitality, Mean (SD | 61.29 (13.77) |
| SF-36 Social Function, Mean (SD | 48.85 (8.60) |
| SF-36 Emotional role limitations, Mean (SD | 16.88 (10.33) |
| SF-36 Mental Health, Mean (SD | 76.83 (15.13) |

BMI = Body Mass Index; SF-36 = Short Form (36) Health Survey

Supplemental table 4 German National Databank of Rheumatic Disease sample characteristics ( N = 4201)

|  |  |
| --- | --- |
| Females, n(%) | 2960 (70.5) |
| Age in years, Mean (SD) | 61.48 (13.95) |
| Swollen joint count, Mean (SD) | 1.03 (2.15) |
| Tender joint count, Mean (SD) | 1.68 (3.44) |
| erythrocyte sedimentation rate, Mean (SD) | 20.5 (17.42) |
| C-reactive protein, Mean (SD) | 0.75 (1.53) |
| NRS patient global assessment wellbeing, Mean (SD) | 3.99 (2.24) |
| NRS physician global assessment, Mean (SD) | 1.83 (1.82) |
| NRS patient global assessment disease activity, Mean (SD) | 3.57 (2.36) |
| Euroqol 5 dimensions , Mean (SD) | 0.79 (0.22) |
| Rheumatoid arthritis impact of disease questionnaire , Mean (SD) | 3.37 (2.23) |

NRS = numerical rating scale

Supplemental table 5 Pharmachild NL sample characteristics (N = 1029)

| Females, n(%) | 775 (64.9%) |
| --- | --- |
| Age in years, Mean (SD) | 16.03 (4.76) |
| VAS pain, Mean (SD) | 22.26 (25.93) |
| VAS general Health, Mean (SD) | 20.34 (25.04) |

VAS = Visual analogue scale

Supplemental table 6 Psoriatic Arthritis Impact of Disease study sample characteristics ( N =474)

| Females, n(%) | 235 (49.6%) |
| --- | --- |
| Age in years, Mean (SD) | 50.38 (12.69) |
| Disease duration in years, Mean (SD) | 9.63 (9.74) |
| Tender joint count in 28 joints, Mean (SD) | 5.07 (13.81) |
| Swollen joint count in 28 joints, Mean (SD) | 3.97 (14.86) |
| Disease Activity Score 28, Mean (SD) | 2.78 (1.42) |
| Euroqol 5 dimensions, Mean (SD) | 0.58 (0.31) |
| Psoriatic Arthritis Impact of Disease score , Mean (SD) | 3.54 (2.34) |
| Sf-36 Physical component score, Mean (SD) | 36.59 (12.04) |
| Sf-36 Mental component score, Mean (SD) | 45.79 (13.59) |

SF-36 = Short Form (36) Health Survey

Supplemental table 7 Swiss Clinical Quality Management sample characteristics ( N = 3157)

| Age in years, Mean (SD) | 1669 (52.9%) |
| --- | --- |
| Disease duration in years, Mean (SD) | 46.60 (14.20) |
| Swollen Joints Count | 4.25 (5.53) |
| Tender joints Count | 6.38 (7.18) |
| Erythrocyte sedimentation rate, Mean (SD) | 20.4 (19.38) |
| C-reactive protein, Mean (SD) | 11.41 (18.12) |
| NRS night pain last 7 days, Mean (SD) | 4.59 (3.03) |
| NRS overall pain last 7 days, Mean (SD) | 5.29 (2.68) |
| NRS patient global assessment disease activity | 5.25 (2.75) |

NRS = numerical rating scale

Supplemental table 8 Items with collapsed response categories

| ITEM code | Item | Collapsed response options |
| --- | --- | --- |
| PROMIS  PFB26 | Are you able to shampoo your hair? | 0) Without any difficulty/ 1) with a little difficulty/ 2) with some difficulty, with much difficulty or Unable to do |
| PROMIS  PFA55 | Are you able to wash and dry your body? | 0) Without any difficulty/ 1) with a little difficulty/ 2) with some difficulty 3) with much difficulty OR Unable to do |
| PROMIS  PFC45r1 | Are you able to sit on and get up from the toilet? | 0) Without any difficulty/ 1) with a little difficulty/ 2) with some difficulty, with much difficulty or Unable to do |
| CHAQ4 | Is your child able to cut fingernails? | 0) Without any difficulty/ 1) with a little difficulty/ 2) with some difficulty 3) with much difficulty OR Unable to do |
| CHAQ5 | Is your child able to stand up from a low chair or floor? | 0) Without any difficulty/ 1) with a little difficulty/ 2) with some difficulty 3) with much difficulty OR Unable to do |
| CHAQ6 | Is your child able to get in and out of bed or stand up in a crib? | 0) Without any difficulty/ 1) with a little difficulty/ 2) with some difficulty 3) with much difficulty OR Unable to do |
| CHAQ7 | Is your child able to cut his/her own meat? | 0) Without any difficulty/ 1) with a little difficulty/ 2) with some difficulty 3) with much difficulty OR Unable to do |
| CHAQ8 | Is your child able to lift a cup or glass to mouth? | 0) Without any difficulty/ 1) with a little difficulty/ 2) with some difficulty 3) with much difficulty OR Unable to do |

Supplemental table 9 differential item functioning across different language versions

| Item | Language DIF Effect size | Language assessed |
| --- | --- | --- |
| BASFI_1 | 0.01 | Swiss, German |
| BASFI_2 | 0.01 | Swiss, German |
| BASFI_3 | 0.02 | Swiss, German |
| BASFI_4 | 0.02 | Swiss, German |
| BASFI_5 | 0.01 | Swiss, German |
| BASFI_6 | 0.02 | Swiss, German |
| BASFI_7 | 0.01 | Swiss, German |
| BASFI_8 | 0.04 | Swiss, German |
| BASFI_9 | 0.03 | Swiss, German |
| BASFI_10 | 0.01 | Swiss, German |
| EQ-5D_1 | 0.01 | Swiss, French, German |
| EQ-5D_2 | 0.01 | Swiss, French, German |
| EQ-5D_3 | 0.01 | Swiss, French, German |
| SF36 PF10_1 | 0.03 | Swiss, Dutch, French, English |
| SF36 PF10_2 | 0.02 | Swiss, Dutch, French, English |
| SF36 PF10_3 | 0.02 | Swiss, Dutch, French, English |
| SF36 PF10_4 | 0.03 | Swiss, Dutch, French, English |
| SF36 PF10_5 | 0.02 | Swiss, Dutch, French, English |
| SF36 PF10_6 | 0.01 | Swiss, Dutch, French, English |
| SF36 PF10_7 | 0.04 | Swiss, Dutch, French, English |
| SF36 PF10_8 | 0.03 | Swiss, Dutch, French, English |
| SF36 PF10_9 | 0.01 | Swiss, Dutch, French, English |
| SF36 PF10_10 | 0.02 | Swiss, Dutch, French, English |
| NRS | 0.01 | French, German |
| HAQ1 | 0.02 | Swiss, Dutch, French, English |
| HAQ2 | 0.01 | Swiss, Dutch, French, English |
| HAQ3 | 0.02 | Swiss, Dutch, French, English |
| HAQ4 | 0.02 | Swiss, Dutch, French, English |
| HAQ5 | 0.02 | Swiss, Dutch, French, English |
| HAQ6 | 0.01 | Swiss, Dutch, French, English |
| HAQ7 | 0.04 | Swiss, Dutch, French, English |
| HAQ8 | 0.01 | Swiss, Dutch, French, English |
| HAQ9 | 0.01 | Swiss, Dutch, French, English |
| HAQ10 | 0.01 | Swiss, Dutch, French, English |
| HAQ11 | 0.04 | Swiss, Dutch, French, English |
| HAQ12 | 0.01 | Swiss, Dutch, French, English |
| HAQ13 | 0.02 | Swiss, Dutch, French, English |
| HAQ14 | 0.02 | Swiss, Dutch, French, English |
| HAQ15 | 0.01 | Swiss, Dutch, French, English |
| HAQ16 | 0.01 | Swiss, Dutch, French, English |
| HAQ17 | 0.01 | Swiss, Dutch, French, English |
| HAQ18 | 0.01 | Swiss, Dutch, French, English |
| HAQ19 | 0.01 | Swiss, Dutch, French, English |
| HAQ20 | 0.02 | Swiss, Dutch, French, English |

BASFI = Bath Ankylosing Spondylitis Function Index; HAQ-DI = Health Assessment PROM Disability Index; NRS = Numerical Rating Scale; SF36 PF10= Short form (36) Health Survey Physical functioning; EQ-5D = Euroqol 5 dimensions

Supplemental figure 2: Item Response models for SF-36 item 10 (Washing and Dressing) before (black lines) and after (grey lines) omitting items flagged for local dependence
